# Variability of adiposity indices and incident heart failure among adults with type 2 diabetes

**DOI:** 10.1186/s12933-021-01440-1

**Published:** 2022-02-01

**Authors:** Arnaud D. Kaze, Sebhat Erqou, Prasanna Santhanam, Alain G. Bertoni, Rexford S. Ahima, Gregg C. Fonarow, Justin B. Echouffo-Tcheugui

**Affiliations:** 1 Department of Medicine, SOVAH Health, Danville, VA USA; 2 Department of Medicine, Division of Cardiology, Providence VA Medical Center and Alpert Medical School of Brown University, Providence, RI USA; 3 Department of Medicine, Division of Endocrinology, Diabetes & Metabolism, Johns Hopkins University School of Medicine, Baltimore, MD 21224 USA; 4 Department of Epidemiology and Prevention, Wake Forest School of Medicine, Winston-Salem, NC USA; 5 Ahmanson-UCLA Cardiomyopathy Center, Ronald Reagan UCLA Medical Center, Los Angeles, CA USA; 6 Welch Prevention Center for Prevention, Epidemiology and Clinical Research, Johns Hopkins University, Baltimore, MD USA

**Keywords:** Cardiovascular disease, Obesity, Diabetes, type 2, Epidemiology, Heart failure

## Abstract

**Background:**

It remains unclear how the variability of adiposity indices relates to incident HF. This study evaluated the associations of the variability in several adiposity indices with incident heart failure (HF) in individuals with type 2 diabetes (T2DM).

**Methods:**

We included 4073 participants from the Look AHEAD (Action for Health in Diabetes) study. We assessed variability of body mass index (BMI), waist circumference (WC), and body weight across four annual visits using three variability metrics, the variability independent of the mean (VIM), coefficient of variation (CV), and intraindividual standard deviation (SD). Multivariable Cox regression models were used to generate adjusted hazard ratios (aHR) and 95% confidence intervals (CI) for incident HF.

**Results:**

Over a median of 6.7 years, 120 participants developed incident HF. After adjusting for relevant confounders including baseline adiposity levels, the aHR for the highest (Q4) versus lowest quartile (Q1) of VIM of BMI was 3.61 (95% CI 1.91–6.80). The corresponding aHRs for CV and SD of BMI were 2.48 (95% CI 1.36–4.53) and 2.88 (1.52–5.46), respectively. Regarding WC variability, the equivalent aHRs were 1.90 (95% CI 1.11–3.26), 1.79 (95% CI 1.07–3.01), and 1.73 (1.01–2.95) for Q4 versus Q1 of VIM, CV and SD of WC, respectively.

**Conclusions:**

In a large sample of adults with T2DM, a greater variability of adiposity indices was associated with higher risks of incident HF, independently of traditional risk factors and baseline adiposity levels.

*Registration-URL:* https://clinicaltrials.gov/ct2/show/NCT00000620.

**Supplementary Information:**

The online version contains supplementary material available at 10.1186/s12933-021-01440-1.

## Background

Type 2 diabetes (T2DM), obesity and heart failure (HF) remain major public health problems across the USA [1–3]. The interplay between these 3 entities is complex; with obesity increasing the risk of HF, and individuals with mild to moderate obesity having a better survival profile compared to those at the extreme adiposity levels [4]. About 12% or more people with diabetes mellitus will develop diabetes-related cardiac dysfunction, which may ultimately lead to overt HF and death [5, 6]. Diabetes-related cardiac dysfunction is thought to occur independently of coronary artery disease (CAD), hypertension, or valvular heart disease. Numerous pathways linking diabetes to cardiac remodeling have been described, and include among others the increased formation of advanced glycation end-products (AGEs), hyperinsulinemia, oxidative stress, autonomic dysfunction and low-grade inflammation [5–7]. The role of inflammation in the pathophysiology of diabetes-related cardiomyopathy is illustrated by evidence of higher plasma concentrations of pro-inflammatory proteomic markers among patients with diabetes-related HF [7].

Major professional organizations recommend the use of weight loss strategies in the management of overweight and obese individuals including those with T2DM [8, 9]. However, weight loss attempts frequently lead to weight cycling, characterized by major fluctuations in body weight [10]. There is accruing evidence that body weight fluctuations are associated with increased risks of atherosclerotic cardiovascular disease (ASCVD) and deaths [11–15]; however, most studies have not explored the relation with incident HF, especially among people with T2DM. Additionally, to our knowledge, the association of variability in waist circumference (WC) with incident HF has never been assessed in T2DM.

Using data from the Look AHEAD (Action for Health in Diabetes) study—a large cohort of adults with T2DM, we evaluated the associations of the variability in adiposity indices (body mass index [BMI], WC, and body weight) with incident HF events. Given the extant evidence on the variability of body composition indices and cardiovascular outcomes other than HF among individuals with diabetes [11–15], as well as the link between glycemic and blood pressure (BP) variability and diabetes-related HF [16, 17], we therefore hypothesized that a higher variability of body composition indices would be related to a heightened risk of incident HF.

## Methods

### Study design

We performed a secondary and prospective cohort analysis of the Look AHEAD study, details of which have previously been published [18]. Briefly, Look AHEAD was a randomized clinical trial in which 5145 overweight or obese adults with T2DM aged 45 to 76 years were recruited from August 2001 to April 2004 across 16 centers in the USA and randomly assigned to receive either an intensive lifestyle intervention (ILI) or diabetes support and education (DSE). For the current analysis, we included participants with full data on body mass index (BMI) and waist circumference (WC) at the baseline, 12-month, 24-month, and 36-month visits. Participants with history of prevalent HF, those who developed HF or died during the initial 36-months (body weight variability assessment period) were excluded (n = 828); this was done in order to ensure that the exposure measurement preceded the development of the outcome. We also excluded participants in the Look AHEAD who had consent restrictions (n = 244). A total of 4073 participants were included in our final analyses. Additional file 1: Fig S1 summarizes the study exclusion process.

The research protocol was reviewed and approved by the Institutional Review Board at each participating clinical center and informed consent was obtained from each participant [18].

### Assessment of variability in adiposity indices

The variability of adiposity indices was assessed during the first 36-months of follow-up in Look AHEAD (Additional file 1: Fig S1). For each participant, height and weight were measured at each visit twice using a stadiometer and digital scale, respectively; the average of the measurements was calculated and used for the analyses. BMI was computed as weight in kilograms divided by the square of height in meters. Waist circumference (WC) was measured using a non-metallic, constant tension tape placed at midlevel between the highest point of the iliac crest and lowest point of the costal border on the mid-axillary line [18].

For each adiposity index (BMI, WC or body weight), variability was defined by three metrics: the intraindividual standard deviation (SD), the coefficient of variation (CV) calculated as 100*SD/mean, and the variability independent of the mean (VIM) calculated as 100*SD/mean^β^ where β represents the regression coefficient based on the natural logarithm of SD on the natural logarithm of the obesity measure’s mean [19].

### Ascertainment of incident heart failure events

Participants were followed from the end of the variability assessment period (36-month visit) through the occurrence of a HF event, death, or end of the study. Semiannual telephone calls and annual visits were conducted. Incident HF events were ascertained by an adjudication committee after reviewing relevant medical records using criteria adapted from the Women’s Health Initiative [20]. Potential cases were grouped into definite or possible acute decompensated HF, chronic stable HF, HF unlikely, or unclassifiable. Incident HF referred to the first hospitalization for definite or possible acute HF exacerbation [21]. Further details about the ascertainment of HF events are provided in Additional file 1: Method S1.

### Covariates

The covariates assessed at baseline include: age, sex, race/ethnicity, randomization arm, duration of diabetes, history of prevalent ASCVD (history of coronary artery disease or stroke at baseline), current smoking, alcohol use, and estimated glomerular filtration rate (eGFR) calculated using the Chronic Kidney Disease Epidemiology Collaboration equation [22]. Additionally, we assessed variables collected during the variability assessment period which include average systolic blood pressure, use of antihypertensive medication, average ratio of total to high-density lipoprotein (HDL) cholesterol, average glycosylated hemoglobin (HbA_1C_) as well as average BMI, WC, and body weight [18]. We also evaluated the variability of other physiologic parameters including SD of blood pressure, heart rate and HbA_1C_.

### Statistical analyses

We compared the characteristics of the participants across quartiles of the VIM of BMI using the χ^2^ test for categorical variables, and the Analysis of Variance or Kruskal–Wallis test for continuous variables.

Incidence rates were calculated as the ratio of the cumulative number of HF events to the total person-years. Person-years were calculated from the end of the variability assessment period to the earliest of HF event, death, or September 14, 2012 (date of trial’s termination).

We used Cox proportional hazards regression models to compute adjusted hazard ratios (HRs) and 95% confidence intervals (CIs) for incident HF. Each adiposity variability index was modelled both as a continuous variable and quartiles using the lowest quartile as reference group.

We built sequential regression models as follows: (1) a first model adjusting for age, sex, race/ethnicity, randomization arm (model 1); (2) a second model adjusted for model 1 variables plus additional adjustment for current smoking, alcohol drinking, use of antihypertensive medication, average systolic blood pressure, average ratio of total to high-density lipoprotein cholesterol, estimated glomerular filtration rate, duration of diabetes, average HbA_1C_, and history of cardiovascular disease (model 2). When variability was assessed using SD or CV, a third model (Model 3) was constructed as model 2 variables with further adjustment for average BMI (when assessing BMI variability), average WC (when evaluating WC variability) or average body weight (when assessing the variability of body weight). In supplementary analyses, we further adjusted for CAD as a time-varying covariate.

In order the assess the effects of the variability in other physiologic variables, we conducted sensitivity analyses adjusting for SD of SBP, heart rate and HbA_1C_.

A two-sided *P*-value of less than 0.05 was considered statistically significant and all analyses were conducted using STATA 14.2 (Stata, Inc, College Station, TX).

## Results

### Characteristics of study participants

The characteristics of participants by quartiles of VIM of BMI are shown in Table 1. Compared to participants in the lowest quartile, those in the highest quartile were younger, more frequently women and more frequently white (and less frequently black). Additionally, participants in the highest quartile had lower HbA_1C_, total-to-HDL cholesterol ratio, average blood pressure measurements and a lower prevalence of ASCVD at baseline. The proportion of participants in the ILI arm increased across increasing quartiles of BMI variability.

**Table 1:** Characteristics of Study Participants by Quartiles of BMI Variability in the Look AHEAD Study

Characteristics	Whole Sample N = 4073	Quartiles of VIM of Body mass index				*P* Value^*^
		Q1 (< 9.46) N = 1016	Q2 (9.46–15.15) N = 1015	Q3 (15.16–23.77) N = 1020	Q4 (> 23.77) N = 1022	
At Baseline						
Age, years	58.9 (6.8)	59.5 (6.9)	58.6 (6.5)	58.9 (6.9)	58.6 (6.7)	0.007
Women, %	59.0	53.2	60.5	62.9	59.6	< 0.001
Race/ethnicity, %						< 0.001
White	67.8	64.9	63.4	68.2	74.5	
Non-Hispanic Black	16.6	19.3	18.6	17.5	11.1	
Hispanic	12.1	11.7	14.4	10.2	11.9	
Randomization arm						< 0.001
Diabetes support and education	49.1	75.6	57.6	38.0	25.0	
Intensive lifestyle intervention	50.9	24.4	42.4	62.0	75.0	
Current smoking, %	3.9	3.1	4.9	4.1	3.6	0.206
Alcohol drinking, %	34.2	37.7	35.5	33.3	30.2	0.002
Duration of diabetes, years	5.0 (2.0–10.0)	5.0 (2.0–10.0)	5.0 (2.0–9.0)	5.0 (2.0–10.0)	5.0 (2.0–10.0)	0.164
eGFR, mL/min/1.73m^2^	90.0 (16.0)	90.2 (15.2	90.2 (16.3	90.4 (16.3	89.2 (16.1	0.288
Prevalent ASCVD	13.1	15.5	13.7	10.2	12.9	0.005
Metabolic equivalents (METs)	7.2 (2.0)	7.5 (2.1)	7.3 (2.0)	7.2 (2.0)	7.0 (1.9)	< 0.001
During variability assessment period						
Mean hemoglobin A_1C_, %	7.0 (1.0)	7.2 (1.0)	7.2 (1.0)	7.0 (1.0)	6.7 (1.0)	< 0.001
Mean total-to-HDL cholesterol ratio	4.2 (1.2)	4.3 (1.1)	4.4 (1.3)	4.2 (1.1)	4.0 (1.1)	< 0.001
Use of BP-lowering drug, %	85.0	85.2	84.4	84.2	86.2	0.559
Mean systolic BP, mm Hg	125.6 (14.1)	126.6 (13.5)	127.3 (14.5)	125.3 (14.0)	123.4 (14.1)	< 0.001
Mean diastolic BP, mm Hg	68.3 (8.0)	69.4 (7.9)	69.2 (8.1)	67.6 (7.8)	66.8 (7.7)	< 0.001
Mean BMI, kg/m^2^	34.8 (5.9)	34.7 (5.5)	35.1 (5.7)	34.7 (5.9)	34.8 (6.3)	0.357
Mean WC, cm	111.1 (13.5)	111.8 (12.4)	111.5 (13.4)	110.6 (13.6)	110.5 (14.6)	0.084
SD of systolic BP, mm Hg	9.6 (4.9)	9.0 (4.6)	9.4 (5.0)	9.7 (4.9)	10.3 (5.0)	< 0.001
SD of diastolic BP, mm Hg	4.8 (2.5)	4.6 (2.3)	4.9 (2.6)	4.8 (2.5)	5.1 (2.4)	< 0.001
SD of heart rate, bpm	6.1 (3.6)	5.4 (3.3)	6.0 (3.4)	5.9 (3.3)	6.8 (4.1)	< 0.001
SD of hemoglobin A_1C_, %	0.5 (0.4)	0.5 (0.4)	0.5 (0.4)	0.6 (0.4)	0.6 (0.4)	< 0.001

Data are mean (SD), median (interquartile range), or proportion as appropriate. *AHEAD* indicates Action for Health in Diabetes, *ASCVD* atherosclerotic cardiovascular disease, *BMI* body mass index; BP, blood pressure, *eGFR* estimated glomerular filtration rate, *HDL* high-density lipoprotein, *Q* quartile, *VIM* variability independent of the mean, *WC* waist circumference

*P value refers to the comparison across VIM quartiles using the chi-square, Analysis of Variance or Kruskal–Wallis tests as appropriate

Over a median follow-up period of 6.7 years (interquartile range 6.0–7.4), 120 participants developed incident HF events (IR per 1000 person-years: 4.6 [95% CI 3.8–5.4]).

### Variability of Body Mass Index

The adjusted HRs of incident HF by BMI variability metrics, evaluated as continuous measures and quartiles, are shown in Table 2. After multivariable adjustment, the HRs for incident HF per unit-SD of the VIM, CV, and SD were 1.21 (95% CI 1.02–1.45), 1.20 (95% CI 1.00–1.44), and 1.16 (95% CI 0.97–1.38) respectively.

**Table 2:** Hazard ratios for incident heart failure by variability of BMI in the look AHEAD study

Measure of Variability	Quartiles of BMI Variability				P trend	Per 1-SD increment
VIM of BMI	< 9.46	9.46–15.15	15.16–23.77	> 23.77	–	–
No Events/No at Risk	20/1016	41/1015	25/1020	34/1022	–	120/4073
Rate/1000 person-years	3.0 (1.9–4.7)	6.3 (4.6–8.5)	3.8 (2.6–5.6)	5.2 (3.7–7.3)	–	4.6 (3.8–5.4)
Model 1	Reference	2.45 (1.44–4.15)†	1.40 (0.77–2.56)	2.00 (1.11–3.59)*	0.138	1.03 (0.85–1.25)
Model 2	Reference	3.00 (1.68–5.37)‡	2.13 (1.10–4.12)*	3.61 (1.91–6.80)‡	0.001	1.21 (1.02–1.45)*
Model 3	NA	NA	NA	NA	NA	NA
CV of BMI, %	< 1.94	1.94–3.10	3.11–4.94	> 4.94	–	–
No Events/No at Risk	23/1016	40/1017	26/1016	31/1024	–	120/4073
Rate/1000 person-years	3.5 (2.3–5.2)	6.1 (4.5–8.3)	3.9 (2.7–5.8)	4.7 (3.3–6.7)	–	4.6 (3.8–5.4)
Model 1	Reference	2.02 (1.22–3.36)†	1.23 (0.69–2.19)	1.47 (0.83–2.63)	0.517	0.98 (0.80–1.20)
Model 2	Reference	2.26 (1.32–3.89)†	1.72 (0.93–3.20)	2.44 (1.33–4.49)†	0.015	1.19 (0.99–1.44)
Model 3	Reference	2.26 (1.32–3.88)†	1.73 (0.94–3.21)	2.48 (1.36–4.53)†	0.013	1.20 (1.00–1.44)
SD of BMI, kg/m^2^	< 0.67	0.67–1.08	1.08–1.70	> 1.70	–	–
No Events/No at Risk	21/1017	36/1015	30/1019	33/1022	–	120/4073
Rate/1000 person-years	3.2 (2.1–4.9)	5.5 (3.9–7.6)	4.6 (3.2–6.6)	5.0 (3.6–7.1)	–	4.6 (3.8–5.4)
Model 1	Reference	2.08 (1.22–3.55)†	1.74 (0.99–3.08)	2.00 (1.12–3.58)*	0.050	1.08 (0.90–1.29)
Model 2	Reference	2.69 (1.50–4.83)†	2.68 (1.44–4.99)†	3.49 (1.86–6.55)‡	< 0.001	1.22 (1.04–1.44)
Model 3	Reference	2.50 (1.39–4.50)†	2.37 (1.27–4.44)†	2.88 (1.52–5.46)†	0.004	1.16 (0.97–1.38)

Data are hazard ratios (95% confidence intervals) unless otherwise indicated

Model 1 adjusted for age, sex, race/ethnicity, and randomization arm

Model 2 includes variables in model 1 with further adjustment for current smoking, alcohol drinking, use of antihypertensive medications, average systolic blood pressure, average ratio of total to high-density lipoprotein cholesterol, average hemoglobin A_1C_, estimated glomerular filtration rate, duration of diabetes, and history of atherosclerotic cardiovascular disease

Model 3 includes model 2 plus further adjustment for average BMI

*AHEAD* indicates Action for Health in Diabetes, *BMI* body mass index, *CV* coefficient of variation, *NA* not applicable, *SD* standard deviation, *VIM* variability independent of the mean

^*^P < 0.05, † *P* < 0.01, ‡ *P* < 0.001

Participants in the top quartile of VIM of BMI had a 3.6-fold higher relative risk of incident HF compared to those in the bottom quartile (HR 3.61, 95% CI 1.91–6.80). The corresponding HRs relating incident HF to the CV, and SD of BMI were 2.48 (95% CI 1.36–4.53), and 2.88 (95% CI 1.52–5.46), respectively.

### Variability of waist circumference

The adjusted HRs for incident HF per SD increment in VIM, CV, and SD of WC were 1.24 (95% CI 1.08–1.42), 1.22 (95% CI 1.05–1.42), and 1.18 (95% CI 1.04–1.35), respectively (Table 3). The adjusted HRs for the highest compared to the lowest quartiles were 1.90 (95% CI 1.11–3.26), 1.79 (95% CI 1.07–3.01), 1.73 (95% CI 1.01–2.95) for VIM, CV, and SD, respectively (Table 3).

**Table 3:** Hazard ratios for incident heart failure by variability of waist circumference in the look AHEAD study

Measure of variability	Quartiles of waist circumference variability				P trend	Per 1-SD increment
VIM of Waist Circumference	< 168.1	168.1–259.8	259.9–392.0	> 392.0	–	–
No Events/No at Risk	28/1019	33/1020	20/1017	39/1017	–	120/4073
Rate/1000 person-years	4.3 (2.9–6.2)	5.0 (3.6–7.0)	3.0 (2.0–4.7)	5.9 (4.3–8.1)		4.6 (3.8–5.4)
Model 1	Reference	1.22 (0.74–2.00)	0.76 (0.43–1.35)	1.46 (0.88–2.41)	0.335	1.14 (0.98–1.33)
Model 2	Reference	1.34 (0.80–2.24)	0.90 (0.49–1.62)	1.90 (1.11–3.26)*	0.072	1.24 (1.08–1.42)†
Model 3	Reference	1.29 (0.77–2.17)	0.87 (0.48–1.58)	1.74 (1.02–2.97)*	0.125	NA
CV of Waist Circumference, %	< 1.87	1.87–2.89	2.90–4.44	> 4.44	–	–
No Events/No at Risk	34/1019	29/1020	19/1018	38/1016	–	120/4073
Rate/1000 person-years	5.2 (3.7–7.3)	4.4 (3.0–6.3)	2.9 (1.8–4.5)	5.8 (4.2–7.9)	–	4.6 (3.8–5.4)
Model 1	Reference	0.90 (0.55–1.47)	0.57 (0.32–1.01)	1.20 (0.74–1.96)	0.732	1.08 (0.91–1.29)
Model 2	Reference	0.97 (0.58–1.61)	0.70 (0.39–1.26)	1.60 (0.95–2.71)	0.210	1.22 (1.03–1.45)*
Model 3	Reference	0.96 (0.57–1.60)	0.76 (0.42–1.36)	1.79 (1.07–3.01)*	0.085	1.22 (1.05–1.42)†
SD of Waist Circumference, cm	< 2.08	2.09–3.22	3.23–4.86	> 4.86	–	–
No Events/No at Risk	28/1019	33/1020	20/1017	39/1017	–	120/4073
Rate/1000 person-years	4.3 (2.9–6.2)	5.0 (3.5–7.0)	3.0 (2.0–4.7)	5.9 (4.3–8.1)	–	4.6 (3.8–5.4)
Model 1	Reference	1.22 (0.74–2.00)	0.76 (0.43–1.35)	1.46 (0.88–2.41)	0.337	1.15 (0.99–1.33)
Model 2	Reference	1.34 (0.80–2.24)	0.90 (0.50–1.63)	1.90 (1.11–3.25)*	0.073	1.24 (1.09–1.42)†
Model 3	Reference	1.29 (0.77–2.16)	0.87 (0.48–1.58)	1.73 (1.01–2.95)*	0.130	1.18 (1.04–1.35)*

Data are hazard ratios (95% confidence intervals) unless otherwise indicated

Model 1 adjusted for age, sex, race/ethnicity, and randomization arm

Model 2 includes variables in model 1 with further adjustment for current smoking, alcohol drinking, use of antihypertensive medications, average systolic blood pressure, average ratio of total to high-density lipoprotein cholesterol, average hemoglobin A_1C_, estimated glomerular filtration rate, duration of diabetes, and history of cardiovascular disease

Model 3 includes model 2 plus further adjustment for average waist circumference

*AHEAD* indicates Action for Health in Diabetes, *CV* coefficient of variation, *SD* standard deviation, *VIM* variability independent of the mean

^*^ P < 0.05, † *P* < 0.01, ‡ *P* < 0.001

### Variability of body weight

Each unit-SD increase in VIM of body weight was associated with higher risks of incident HF (HR 1.22, 95% CI 1.02–1.45, Additional file 1: Table S1). Participants in the top quartile of VIM of body weight had a higher risk of incident HF compared to those in the bottom quartile (HR 2.79, 95% CI 1.50–5.19). The HRs for the top compared to the bottom quartiles were 2.52 (95% CI 1.38–4.62), and 2.00 (95% CI 1.09–3.66) for CV and SD of body weight, respectively (Additional file 1: Table S1).

### Additional analyses

In additional analyses, we performed further adjustments for CAD modelled as a time-varying covariate. These did not materially affect the magnitude or significance of the results (Additional file 1: Tables S2, S3 & S4). Moreover, we tested for the VIM of BMI by various prespecified subgroups statistical interactions. We did not observe any interaction with age (*P* for interaction = 0.947), sex (*P* for interaction = 0.533), and randomization arm (*P* for interaction = 0.402).

In order to evaluate the effects of the variability in other physiologic variables, we conducted sensitivity analyses accounting for SD of SBP, heart rate and HbA_1C_. The magnitude and significance of our findings remained essentially unchanged (Additional file 1: Table S5).

## Discussion

We conducted a comprehensive evaluation of the associations between variability of adiposity indices and incident HF in a large sample of adults with T2DM. We found that variability of BMI, WC and body weight were each independently associated with an increased risk of incident HF, after adjusting for other known HF risk factors including baseline adiposity levels. Our results were consistent across several variability metrics and were independent of incident CAD. Our findings underscore the importance of minimizing weight fluctuations during weight loss attempts among overweight or obese patients with T2DM.

Our study is unique in several ways including its assessment of the variability of waist circumference as prior studies typically focused on BMI, a measure of global obesity which does not capture the distribution of adiposity in the body [12–14, 23, 24]. Additionally, this study complements the available body of evidence by focusing on the effect of adiposity indices’ variability on incident HF in individuals with T2DM. The limited number of studies on this topic focused on atherosclerotic cardiovascular outcomes [12–14, 24]. The positive association between body weight variability and HF observed in this study is consistent with prior reports which found that body weight variability was positively associated with CVD events and deaths [12–14, 24, 25].

While the pathways relating body weight variability to a higher risk of HF in T2DM are not entirely known, possible hypotheses include the hypertrophy of adipose tissue resulting from metabolic shifts that follow weight cycling [26]. Furthermore, body weight fluctuations are positively associated with greater incidence of the metabolic syndrome components which might in turn augment the risk of HF [25, 27, 28]. Another possible mechanism is via the effect of low-grade inflammation as documented by higher concentrations of plasma C-reactive protein and the increased number of CD4 and CD8 T lymphocytes with increased production of cytokines in those who have higher degree of weight cycling [29, 30].

The public health and research implications of our findings are manifold for people with T2DM. The prevention of body weight fluctuations during weight loss attempts should be a priority and may help reduce the high burden of HF hospitalizations in people with T2DM on the healthcare system. Moreover, further studies are needed to explore the pathways linking body weight variability to HF.

Our study has several strengths. First, we evaluated a vast array of adiposity measures, including WC variability, which was not always included in prior studies. Second, our study includes a large and racially diverse sample of individuals with T2DM, with a standardized assessment of anthropometric measures at regular predetermined intervals (thus allowing the variability assessment), a blinded adjudication of outcomes, a long duration of follow-up, and the consistency of the observed associations with the risk of HF across measures of variability.

Our results should be interpreted in the context of a few limitations. First, we relied only on four timepoints to measure variability; thus, we may have underestimated variability, as previously suggested by data from the blood pressure variability literature [31]. Second, our analysis was observational; therefore, we cannot establish causal association between adiposity variability and HF, and residual confounding may exist. Additionally, we did not have data on the frequency (prevalence and incidence) of malignancies, which may affect weight cycling. Hence, we could not account for the presence of malignancies in our analyses. Third, we did not have data on left ventricular ejection fraction, which would have allowed the identification of HF subtypes. Thus, we could not assess the associations between variability in adiposity indices and the risk of incident HF with reduced ejection fraction and HF with preserved ejection fraction. Fourth, we did not have data on physical activity and dietary intake collected on the entire cohort of Look AHEAD participants; hence we could not evaluate the interplay between those variables, variability in adiposity indices, and incident HF.

## Conclusions

In summary, in a large sample of adults with T2DM, a greater variability of BMI, waist circumference, and body weight was associated with higher risk of incident HF, above and beyond baseline adiposity levels. Our findings underscore the need to avoid excessive weight fluctuations during weight loss attempts in this high-risk population.

## Supplementary Information

**Additional file 1: Fig S1**. Study design. **Table S1**. Hazard Ratios for Incident Heart Failure by Variability of Body Weight in the Look AHEAD Study. **Table S2**. Hazard Ratios for Incident Heart Failure by Variability of BMI in the Look AHEAD Study. **Table S3**. Hazard Ratios for Incident Heart Failure by Variability of Waist Circumference in the Look AHEAD Study. **Table S4**. Hazard Ratios for Incident Heart Failure by Variability of Body Weight in the Look AHEAD Study. **Table S5**. Hazard Ratios for Incident Heart Failure after additional adjustments for Variability in Other physiologic parameters.

## Data Availability

The data that support the findings of this study are available from the NIDDK Central Repositories, but restrictions apply to the availability of these data. Authors’ Note: (1) The Look AHEAD study was conducted by the Look AHEAD Investigators and supported by the National Institute of Diabetes and Digestive and Kidney Diseases (NIDDK). The data from the Look AHEAD reported here were supplied by the NIDDK Central Repositories. This manuscript was not prepared in collaboration with Investigators of the Look AHEAD study and does not necessarily reflect the opinions or views of the Look Ahead Study, the NIDDK Central Repositories, or the NIDDK. (2) Look AHEAD was conducted by the Look AHEAD Research Group and supported by the National Institute of Diabetes and Digestive and Kidney Diseases (NIDDK); the National Heart, Lung, and Blood Institute (NHLBI); the National Institute of Nursing Research (NINR); the National Institute of Minority Health and Health Disparities (NIMHD); the Office of Research on Women's Health (ORWH); and the Centers for Disease Control and Prevention (CDC). The data (and samples) from Look AHEAD were supplied by the NIDDK Central Repositories. This manuscript was not prepared under the auspices of the Look AHEAD and does not represent analyses or conclusions of the Look AHEAD Research Group, the NIDDK Central Repositories, or the NIH.

## Abbreviations

AHEAD: Action for Health in Diabetes
ASCVD: Atherosclerotic cardiovascular disease
BP: Blood pressure
BMI: Body mass index
CAD: Coronary artery disease
CI: Confidence interval
CVD: Cardiovascular disease
DSE: Diabetes support and education
eGFR: Estimated glomerular filtration rate
HbA_1C_: Hemoglobin A_1C_
HDL: High-density lipoprotein
HF: Heart failure
HR: Hazard ratio
ILI: Intensive lifestyle intervention
NIDDK: National Institute of Diabetes and Digestive and Kidney Diseases
IR: Incidence rate
WC: Waist circumference

## References

[1] Menke A, Casagrande S, Geiss L, Cowie CC (2015). Prevalence of and trends in diabetes among adults in the United States, 1988–2012. JAMA.

[2] Virani SS, Alonso A, Aparicio HJ, Benjamin EJ, Bittencourt MS, Callaway CW (2021). Heart disease and stroke statistics-2021 update: a report from the American Heart Association. Circulation.

[3] Flegal KM, Kruszon-Moran D, Carroll MD, Fryar CD, Ogden CL (2016). Trends in obesity among adults in the United States, 2005 to 2014. JAMA.

[4] Horwich TB, Fonarow GC, Clark AL (2018). Obesity and the obesity paradox in heart failure. Prog Cardiovasc Dis.

[5] Lorenzo-Almorós A, Tuñón J, Orejas M, Cortés M, Egido J, Lorenzo Ó (2017). Diagnostic approaches for diabetic cardiomyopathy. Cardiovasc Diabetol.

[6] Cong S, Ramachandra CJA, Mai Ja KM, Yap J, Shim W, Wei L (2020). Mechanisms underlying diabetic cardiomyopathy: from pathophysiology to novel therapeutic targets. Cond Med.

[7] Verdonschot JAJ, Ferreira JP, Pellicori P, Brunner-La Rocca H-P, Clark AL, Cosmi F (2021). Proteomic mechanistic profile of patients with diabetes at risk of developing heart failure: insights from the HOMAGE trial. Cardiovasc Diabetol.

[8] American Diabetes Association (2020). 8. Obesity management for the treatment of type 2 diabetes: standards of medical care in diabetes-2020. Diabetes Care.

[9] Jensen MD, Ryan DH, Apovian CM, Ard JD, Comuzzie AG, Donato KA (2014). 2013 AHA/ACC/TOS guideline for the management of overweight and obesity in adults: a report of the American College of Cardiology/American Heart Association Task Force on Practice Guidelines and The Obesity Society. J Am Coll Cardiol.

[10] Montani J-P, Schutz Y, Dulloo AG (2015). Dieting and weight cycling as risk factors for cardiometabolic diseases: who is really at risk?. Obes Rev an Off J Int Assoc Study Obes.

[11] Lissner L, Odell PM, D’Agostino RB, Stokes J, Kreger BE, Belanger AJ (1991). Variability of body weight and health outcomes in the Framingham population. N Engl J Med.

[12] Nam GE, Kim W, Han K, Lee C-W, Kwon Y, Han B (2020). Body weight variability and the risk of cardiovascular outcomes and mortality in patients with type 2 diabetes: a Nationwide Cohort Study. Diabetes Care.

[13] Bangalore S, Fayyad R, DeMicco DA, Colhoun HM, Waters DD (2018). Body weight variability and cardiovascular outcomes in patients with type 2 diabetes mellitus. Circ Cardiovasc Qual Outcomes.

[14] Yeboah P, Hsu F-C, Bertoni AG, Yeboah J (2019). Body mass index, change in weight, body weight variability and outcomes in type 2 diabetes mellitus (from the ACCORD Trial). Am J Cardiol.

[15] Bangalore S, Fayyad R, Laskey R, DeMicco DA, Messerli FH, Waters DD (2017). Body-weight fluctuations and outcomes in coronary disease. N Engl J Med.

[16] Segar MW, Patel KV, Vaduganathan M, Caughey MC, Butler J, Fonarow GC (2020). Association of long-term change and variability in glycemia with risk of incident heart failure among patients with type 2 diabetes: a secondary analysis of the ACCORD Trial. Diabetes Care.

[17] Kaze AD, Santhanam P, Erqou S, Bertoni AG, Ahima RS, Echouffo-Tcheugui JB (2021). Long-term variability of blood pressure and incidence of heart failure among individuals with type 2 diabetes. ESC Hear Fail.

[18] Ryan DH, Espeland MA, Foster GD, Haffner SM, Hubbard VS, Johnson KC, et al. Look AHEAD (Action for Health in Diabetes): design and methods for a clinical trial of weight loss for the prevention of cardiovascular disease in type 2 diabetes. Control Clin Trials 2003;24:610–28. https://www.ncbi.nlm.nih.gov/pubmed/14500058. 10.1016/s0197-2456(03)00064-3 14500058

[19] Echouffo-Tcheugui JB, Zhao S, Brock G, Matsouaka RA, Kline D, Joseph JJ (2019). Visit-to-visit glycemic variability and risks of cardiovascular events and all-cause mortality: the ALLHAT Study. Diabetes Care.

[20] Hall PS, Nah G, Howard BV, Lewis CE, Allison MA, Sarto GE (2017). Reproductive factors and incidence of heart failure hospitalization in the women’s health initiative. J Am Coll Cardiol.

[21] Pandey A, Patel KV, Bahnson JL, Gaussoin SA, Martin CK, Balasubramanyam A (2020). Association of intensive lifestyle intervention, fitness and body mass index with risk of heart failure in overweight or obese adults with type 2 diabetes mellitus: an analysis from the look AHEAD trial. Circulation.

[22] Levey AS, Stevens LA, Schmid CH, Zhang YL, Castro AF, Feldman HI (2009). A new equation to estimate glomerular filtration rate. Ann Intern Med.

[23] Després J-P (2012). Body fat distribution and risk of cardiovascular disease: an update. Circulation.

[24] Zoppini G, Verlato G, Targher G, Bonora E, Trombetta M, Muggeo M (2008). Variability of body weight, pulse pressure and glycaemia strongly predict total mortality in elderly type 2 diabetic patients. The Verona Diabetes Study. Diabetes Metab Res Rev.

[25] Kim MK, Han K, Park Y-M, Kwon H-S, Kang G, Yoon K-H (2018). Associations of variability in blood pressure, glucose and cholesterol concentrations, and body mass index with mortality and cardiovascular outcomes in the general population. Circulation.

[26] Cereda E, Malavazos AE, Caccialanza R, Rondanelli M, Fatati G, Barichella M (2011). Weight cycling is associated with body weight excess and abdominal fat accumulation: a cross-sectional study. Clin Nutr.

[27] Lissner L, Andres R, Muller DC, Shimokata H (1990). Body weight variability in men: metabolic rate, health and longevity. Int J Obes.

[28] Zhang H, Tamakoshi K, Yatsuya H, Murata C, Wada K, Otsuka R (2005). Long-term body weight fluctuation is associated with metabolic syndrome independent of current body mass index among Japanese men. Circ J.

[29] Tamakoshi K, Yatsuya H, Kondo T, Ishikawa M, Zhang H, Murata C (2003). Long-term body weight variability is associated with elevated C-reactive protein independent of current body mass index among Japanese men. Int J Obes Relat Metab Disord J Int Assoc Study Obes..

[30] Anderson EK, Gutierrez DA, Kennedy A, Hasty AH (2013). Weight cycling increases T-cell accumulation in adipose tissue and impairs systemic glucose tolerance. Diabetes.

[31] Levitan EB, Kaciroti N, Oparil S, Julius S, Muntner P (2012). Blood pressure measurement device, number and timing of visits, and intra-individual visit-to-visit variability of blood pressure. J Clin Hypertens (Greenwich).

