# Supplementary material for: Variability of adiposity indices and incident heart failure among adults with type 2 diabetes
Source: Cardiovasc Diabetol. 2022 Feb 1;21:16. doi: 10.1186/s12933-021-01440-1 (PMC8805255; doi:10.1186/s12933-021-01440-1)
Supplement: Supplementary file 1 — Additional file 1: Fig S1. Study design. Table S1. Hazard Ratios for Incident Heart Failure by Variability of Body Weight in the Look AHEAD Study. Table S2. Hazard Ratios for Incident Heart Failure by Variability of BMI in the Look AHEAD Study. Table S3. Hazard Ratios for Incident Heart Failure by Variability of Waist Circumference in the Look AHEAD Study. Table S4. Hazard Ratios for Incident Heart Failure by Variability of Body Weight in the Look AHEAD Study. Table S5. Hazard Ratios for Incident Heart Failure after additional adjustments for Variability in Other physiologic parameters. [file 12933_2021_1440_MOESM1_ESM.docx]

**DATA SUPPLEMENT**

**Variability of Adiposity Indices and Incident Heart Failure among Adults with Type 2 Diabetes**


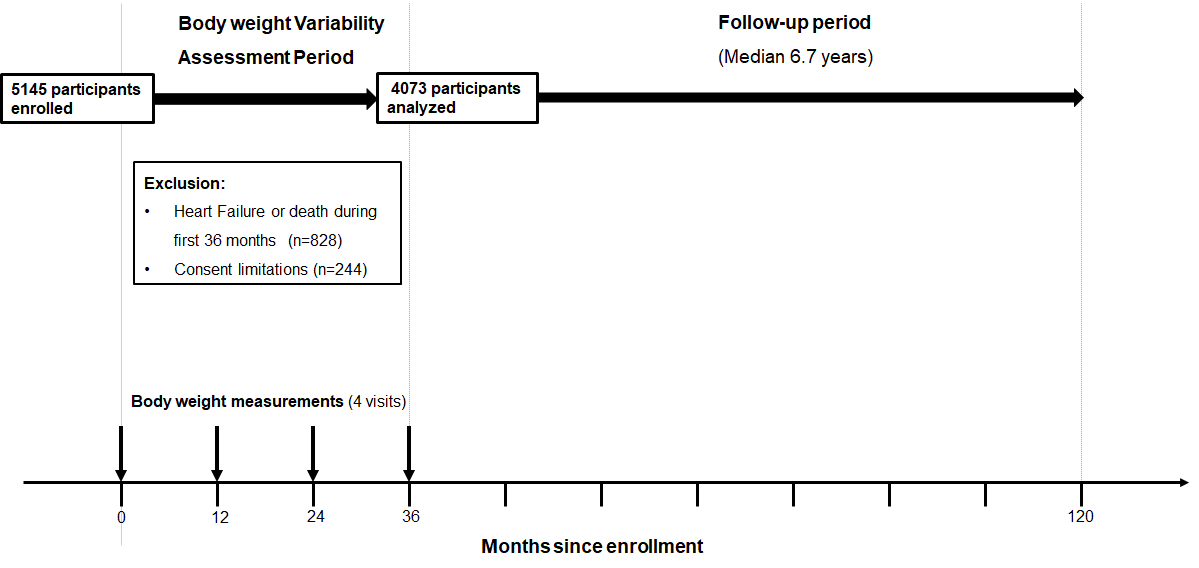


**Supplementary Fig S1.** Study design

**Supplementary Method S1**

Criteria for hospitalized heart failure (HF) were adapted from the Women’s Health Initiative, and did not include cardiogenic shock complicating MI. HF was adjudicated when the participant was hospitalized for new onset or worsened heart failure. Criteria included physician diagnosis of HF, favoring discharge over admitting diagnosis, and medical therapy for HF on admission (eg, diuretics), past medical history documenting previous imaging procedure showing impaired systolic or diastolic left ventricular function, pulmonary edema or congestion on chest x-ray during admission, and/or documentation of imaging during the admission showing dilated or poor left- or right-sided ventricular function or evidence of left ventricular diastolic dysfunction.

**Supplementary Table S1. Hazard Ratios for Incident Heart Failure by Variability of Body Weight in the Look AHEAD Study**

| **Measure of Variability** | **Quartiles of Body Weight Variability** | | | | **P trend** | **Per SD** |
| --- | --- | --- | --- | --- | --- | --- |
| VIM of Body weight | Q1 | Q2 | Q3 | Q4 |  |  |
| Model 1 | Reference | 2.08 (1.23-3.53)† | 1.54 (0.87-2.74) | 1.73 (0.96-3.10) | 0.187 | 1.04 (0.86-1.26) |
| Model 2 | Reference | 2.36 (1.34-4.15)† | 2.16 (1.16-4.01)*** | 2.79 (1.50-5.19)† | 0.003 | 1.22 (1.02-1.45)*** |
| Model 3 | Reference | 2.35 (1.34-4.14)† | 2.04 (1.10-3.79)*** | 2.59 (1.40-4.80)† | 0.008 | 1.19 (0.99-1.42) |
| CV of Body weight |  |  |  |  |  |  |
| Model 1 | Reference | 2.12 (1.27-3.55)† | 1.30 (0.72-2.33) | 1.57 (0.87-2.82) | 0.404 | 0.99 (0.81 -1.22) |
| Model 2 | Reference | 2.26 (1.32-3.89)† | 1.73 (0.93-3.21) | 2.45 (1.33-4.50)† | 0.015 | 1.19 (0.99-1.45) |
| Model 3 | Reference | 2.27 (1.32-3.89)† | 1.76 (0.95-3.26) | 2.52 (1.38-4.62)† | 0.011 | 1.20 (1.00-1.45) |
| SD of Body weight |  |  |  |  |  |  |
| Model 1 | Reference | 1.69 (1.00-2.86) | 1.38 (0.79-2.43) | 1.68 (0.96-2.94) | 0.146 | 1.09 (0.91-1.30) |
| Model 2 | Reference | 1.71 (0.97-2.99) | 1.83 (1.01-3.33)*** | 2.44 (1.35-4.41)† | 0.004 | 1.24 (1.05-1.45)*** |
| Model 3 | Reference | 1.62 (0.92-2.84) | 1.59 (0.87-2.90) | 2.00 (1.09-3.66)*** | 0.039 | 1.17 (0.98-1.39) |

Data are hazard ratios (95% confidence intervals) unless otherwise indicated.

Model 1 adjusted for age, sex, race/ethnicity, and randomization arm.

Model 2 includes variables in model 1 with further adjustment for current smoking, alcohol drinking, use of antihypertensive medications, average systolic blood pressure, average ratio of total to high-density lipoprotein cholesterol, average hemoglobin A_1C_, estimated glomerular filtration rate, duration of diabetes, and history of cardiovascular disease.

Model 3 includes model 2 plus further adjustment for average body weight.

AHEAD indicates Action for Health in Diabetes; CV, coefficient of variation; SD, standard deviation; VIM, variability independent of the mean; Q, quartile.

** P<*0.05, † *P<*0.01, ‡ *P<*0.001.

**Supplementary Table S2. Hazard Ratios for Incident Heart Failure by Variability of BMI in the Look AHEAD Study**

| **Measure of Variability** | **Quartiles of BMI Variability** | | | | **P trend** | **Per 1-SD increment** |
| --- | --- | --- | --- | --- | --- | --- |
| **VIM of BMI** | **< 9.46** | **9.46-15.15** | **15.16-23.77** | **> 23.77** | … | … |
|  | Reference | 3.21 (1.76-5.85)‡ | 2.18 (1.11-4.29)* | 3.61 (1.88-6.93)‡ | 0.001 | 1.22 (1.02-1.46)* |
| **CV of BMI, %** | **< 1.94** | **1.94-3.10** | **3.11-4.94** | **> 4.94** | … | … |
|  | Reference | 2.37 (1.36-4.14)† | 1.93 (1.03-3.62)* | 2.72 (1.46-5.06)† | 0.006 | 1.24 (1.04-1.49)* |
| **SD of BMI, kg/m^2^** | **< 0.67** | **0.67-1.08** | **1.08-1.70** | **> 1.70** | … | … |
|  | Reference | 2.69 (1.48-4.92)† | 2.42 (1.27-4.61)† | 3.17 (1.64-6.12)† | 0.002 | 1.20 (1.01-1.42)* |

Data are hazard ratios (95% confidence intervals) unless otherwise indicated.

Hazard ratios are adjusted for age, sex, race/ethnicity, and randomization arm, current smoking, alcohol drinking, use of antihypertensive medications, average systolic blood pressure, average ratio of total to high-density lipoprotein cholesterol, average hemoglobin A_1C_, estimated glomerular filtration rate, duration of diabetes, and history of atherosclerotic cardiovascular disease, average BMI and incident coronary artery disease as a time-varying covariate.

AHEAD indicates Action for Health in Diabetes; BMI, body mass index; CV, coefficient of variation; NA, not applicable; SD, standard deviation; VIM, variability independent of the mean.

** P<*0.05, † *P<*0.01, ‡ *P<*0.001.

**Supplementary Table S3. Hazard Ratios for Incident Heart Failure by Variability of Waist Circumference in the Look AHEAD Study**

| **Measure of Variability** | **Quartiles of Waist Circumference Variability** | | | | **P trend** | **Per 1-SD increment** |
| --- | --- | --- | --- | --- | --- | --- |
| **VIM of Waist Circumference** | **< 168.1** | **168.1-259.8** | **259.9-392.0** | **> 392.0** | … | … |
|  | Reference | 1.31 (0.78-2.20) | 0.84 (0.46-1.54) | 1.84 (1.07-3.16)* | 0.100 | 1.21 (1.05-1.38)† |
| **CV of Waist Circumference, %** | **< 1.87** | **1.87-2.89** | **2.90-4.44** | **> 4.44** | … | … |
|  | Reference | 1.01 (0.60-1.69) | 0.74 (0.41-1.33) | 1.90 (1.13-3.21)* | 0.070 | 1.24 (1.07-1.45)† |
| **SD of Waist Circumference, cm** | **< 2.08** | **2.09-3.22** | **3.23-4.86** | **> 4.86** | … | … |
|  | Reference | 1.31 (0.78-2.21) | 0.84 (0.46-1.54) | 1.83 (1.07-3.14)* | 0.105 | 1.20 (1.05-1.38)† |

Data are hazard ratios (95% confidence intervals) unless otherwise indicated.

Hazard ratios are adjusted for age, sex, race/ethnicity, and randomization arm, smoking, alcohol drinking, use of antihypertensive medications, average systolic blood pressure, average ratio of total to high-density lipoprotein cholesterol, average hemoglobin A_1C_, estimated glomerular filtration rate, duration of diabetes, and history of cardiovascular disease, average waist circumference, and incident coronary artery disease as a time-varying covariate.

AHEAD indicates Action for Health in Diabetes; CV, coefficient of variation; SD, standard deviation; VIM, variability independent of the mean.

** P<*0.05, † *P<*0.01, ‡ *P<*0.001.

**Supplementary Table S4. Hazard Ratios for Incident Heart Failure by Variability of Body Weight in the Look AHEAD Study**

| **Measure of Variability** | **Quartiles of Body Weight Variability** | | | | **P trend** | **Per SD** |
| --- | --- | --- | --- | --- | --- | --- |
| VIM of Body weight | Q1 | Q2 | Q3 | Q4 |  |  |
|  | Reference | 2.65 (1.48-4.74)† | 2.17 (1.15-4.10)* | 2.90 (1.54-5.46)† | 0.004 | 1.24 (1.04-1.48)* |
| CV of Body weight | Q1 | Q2 | Q3 | Q4 |  |  |
|  | Reference | 2.40 (1.37-4.18)† | 1.97 (1.05-3.71)* | 2.77 (1.49-5.17)† | 0.005 | 1.25 (1.04-1.51)* |
| SD of Body weight | Q1 | Q2 | Q3 | Q4 |  |  |
|  | Reference | 1.68 (0.94-2.98) | 1.61 (0.87-3.00) | 2.23 (1.20-4.14)* | 0.019 | 1.22 (1.02-1.45)* |

Data are hazard ratios (95% confidence intervals) unless otherwise indicated.

Hazard ratios are adjusted for age, sex, race/ethnicity, and randomization arm, current smoking, alcohol drinking, use of antihypertensive medications, average systolic blood pressure, average ratio of total to high-density lipoprotein cholesterol, average hemoglobin A_1C_, estimated glomerular filtration rate, duration of diabetes, and history of cardiovascular disease, average body weight, and incident coronary artery disease as a time-varying covariate.

AHEAD indicates Action for Health in Diabetes; CV, coefficient of variation; SD, standard deviation; VIM, variability independent of the mean; Q, quartile.

** P<*0.05, † *P<*0.01, ‡ *P<*0.001.

**Supplementary Table S5. Hazard Ratios for Incident Heart Failure after additional adjustments for Variability in Other physiologic parameters**

| **Measure of Variability** | **Hazard ratios (95% CI)** | | | | **P trend** | **Per 1-SD increment** |
| --- | --- | --- | --- | --- | --- | --- |
| **Variability of body mass index** | | | | | | |
| **VIM of BMI** | Reference | 2.93 (1.61-5.35)‡ | 2.13 (1.08-4.19)* | 3.44 (1.77-6.68)‡ | 0.002 | 1.18 (0.97-1.44) |
| **CV of BMI** | Reference | 2.20 (1.26-3.84)† | 1.74 (0.93-3.27) | 2.32 (1.24-4.38)† | 0.030 | 1.17 (0.95-1.43) |
| **SD of BMI** | Reference | 2.42 (1.32-4.42)† | 2.28 (1.20-4.34)* | 2.68 (1.37-5.24)† | 0.012 | 1.13 (0.93-1.37) |
| **Variability of Waist circumference** | | | | | | |
| **VIM of WC** | Reference | 1.31 (0.78-2.20) | 0.85 (0.47-1.55) | 1.80 (1.04-3.11)* | 0.120 | 1.24 (1.07-1.42)† |
| **CV of WC** | Reference | 0.93 (0.56-1.56) | 0.74 (0.41-1.32) | 1.70 (1.00-2.88) | 0.135 | 1.22 (1.05-1.43)* |
| **SD of WC** | Reference | 1.26 (0.75-2.11) | 0.83 (0.46-1.52) | 1.67 (0.97-2.86) | 0.177 | 1.19 (1.03-1.36)* |

Hazard ratios were obtained from Cox proportional hazards models adjusted for age, sex, race/ethnicity, randomization arm, current smoking, alcohol drinking, use of antihypertensive medications, average systolic blood pressure, average ratio of total to high-density lipoprotein cholesterol, average hemoglobin A_1C_, estimated glomerular filtration rate, duration of diabetes, history of atherosclerotic cardiovascular disease, SD of systolic blood pressure, SD of hemoglobin A_1C_, SD of heart rate, and average BMI (or average WC). For VIM, average BMI (or WC) were not included in the models.

BMI indicates body mass index; CI, confidence interval; CV, coefficient of variation; SD, standard deviation; VIM, variability independent of the mean; WC, waist circumference

** P<*0.05, † *P<*0.01, ‡ *P<*0.001.
